# Rapid Recovery From Cocaine-Induced Cardiomyopathy: A Case Report

**DOI:** 10.7759/cureus.49793

**Published:** 2023-12-01

**Authors:** Ibrahim Kamel, Amr Saleh, Sadaf Esteghamati, Harold Dietzius

**Affiliations:** 1 Internal Medicine, Steward Carney Hospital, Dorchester, USA; 2 Internal Medicine, Yale University, New Haven, USA; 3 Internal Medicine, University of La Verne, La Verne, USA; 4 Cardiology, Steward Carney Hospital, Dorchester, USA

**Keywords:** farxiga, cocaine use, reduced ejection fraction, heart failure, cocaine-induced cardiomyopathy

## Abstract

A 33-year-old male presented with shortness of breath and altered mental status. The urine toxicology test was positive for cocaine and fentanyl. The patient underwent a 2D echocardiogram showing severely reduced ejection fraction (EF) and global hypokinesia. He was diagnosed with cocaine-induced cardiomyopathy, which markedly improved four days later.

## Introduction

This case report delves into the intriguing clinical scenario of a 33-year-old male presenting with shortness of breath and altered mental status, ultimately diagnosed with cocaine-induced cardiomyopathy. The report unfolds the patient's rapid recovery within four days of medical intervention, shedding light on the unique aspects of cocaine-related cardiac complications. This not only accentuates the urgency in understanding and managing cocaine-induced cardiomyopathy but also underscores the critical need for well-defined diagnostic criteria and comprehensive management guidelines in addressing this complex medical condition. In 2016, the total number of cocaine users was estimated to be 18.2 million worldwide [1]. Approximately 34% of these cocaine users resided in North America, and 20% resided in Western and Central Europe. In the US, there were 1.5 million cocaine users aged 12 or older, representing 0.6% of the population [2].

## Case presentation

A 33-year-old male was admitted due to shortness of breath and altered mental status (AMS). In our initial physical examination, the patient was confused, and somnolent. His vital signs: heart rate 117 bpm, blood pressure 94/45, respiratory rate 26, oxygen saturation 84 on 5L oxygen, and body temperature 98.5 F. Chest auscultation was remarkable for wheezing and crackles on bilateral lung bases. The urine toxicology screening test was positive for cocaine and fentanyl. Serum alcohol was negative.

Past medical history

The patient had a past medical history of asthma and cocaine abuse.

Differential diagnosis

Based on our preliminary observation, cocaine-induced cardiomyopathy (CIC), acute myocardial infarction (MI), aspiration pneumonia, and pulmonary embolism (PE) were among our possible diagnoses.

Investigations

**Figure 1:**
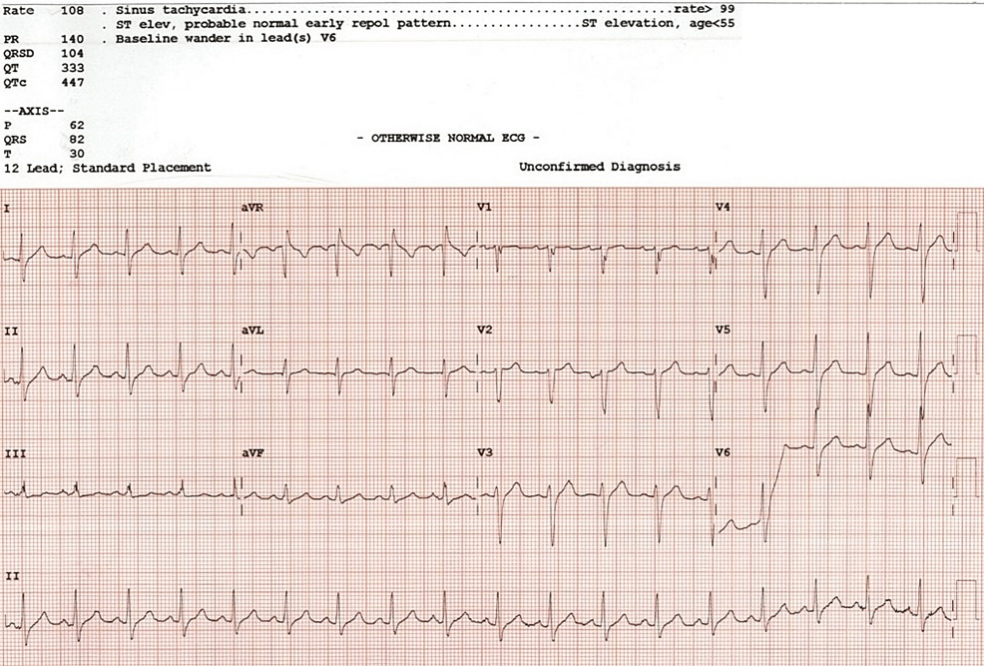
EKG remarkable for sinus tachycardia with early repolarization pattern

The chest X-ray (CXR) on the day of admission revealed multifocal bilateral lung infiltrations. The electrocardiogram (ECG) showed sinus tachycardia with a ventricular rate of 108 with ST-segment elevations due to an early repolarization pattern (Figure 1).

Initial complete blood count (CBC) showed a white blood cell (WBC) count of 19.9 × 103/µL. Venous blood gas (VBG) and comprehensive metabolic panel (CMP) suggest lactic acidosis. The estimated glomerular filtration rate (eGFR) was 21 mL/min/1.73 m^2^. Troponin T was elevated at 846 ng/L. Total creatine kinase (CK) was 4810 U/L. N-terminal pro-b-type natriuretic peptide (NT-proBNP) was 401 pg/mL (normal). The chest computed tomography scan (CT scan) was significant for multifocal bilateral patchy infiltrates involving predominantly the posterior segment of bilateral upper and lower lobes, concerning for aspiration. We also performed a brain CT scan, which showed no evidence of acute intracranial pathology. The 2D echo on the day of admission revealed a calculated ejection fraction (EF) of 22% and a mildly elevated pulmonary artery systolic pressure (37-45 mmHg). Mild right and left atrial enlargement, right ventricular enlargement, global left ventricular hypokinesis, and left ventricular hypertrophy were also reported. Left ventricular systolic function was severely reduced while right ventricular systolic function was moderately to markedly reduced. A possible McConnell's sign (hypokinesis of the right ventricular sparing the apex) was reported, suggestive of pulmonary embolism as the cause of right ventricular dysfunction (Video 1).

**Video 1:** 2D echo four-chamber view with an EF of 22% EF: ejection fraction

Management 

Given the patient's altered mental status and hypoxic respiratory failure, he was admitted to the intensive care unit (ICU). The working diagnosis for the patient's AMS was toxic metabolic encephalopathy. 

As previously mentioned, the urine toxicology screening test revealed positive results for cocaine and fentanyl. Considering these findings, along with the results of the 2D echocardiogram, our provisional diagnosis leaned toward cocaine-induced cardiomyopathy, although we couldn't entirely rule out ischemic causes. The patient also presented with acute kidney injury (AKI) linked to rhabdomyolysis. In the ICU, the patient received fluid resuscitation, but concerns about pulmonary edema arose due to increased oxygen requirements. As a result, the patient was transitioned to IV lasix, which effectively alleviated the dyspnea and reduced oxygen requirements.

An ischemic workup and chest computed tomography angiography (CTA) were recommended when the patient's condition improved. A venous duplex revealed no evidence of deep vein thrombosis. By the third day of admission, the patient's mental status returned to baseline, showing significant improvement, and laboratory values were largely within normal limits. This improvement allowed us to investigate further, although a CTA was ruled out due to the patient's iodine allergy.

On the fourth day of admission, a myocardial perfusion scan was performed, yielding negative results for infarct or reversible ischemia. The reported ejection fraction was 47%. A subsequent 2D echo indicated a remarkable global improvement in cardiac function, with a calculated ejection fraction of 44% (Video 2).

**Video 2:** 2D echo four-chamber view with an EF of 44% EF: ejection fraction

Some regional wall motion abnormalities were still present. He was started on Farxiga 10 mg/day. After the improvement of the patient's symptoms, he was discharged home.

## Discussion

It is challenging to quantify the incidence of cardiac ischemia secondary to cocaine exposure. In a retrospective study of 2097 individuals ≤50 years with type 1 acute myocardial infarction (MI) between 2000 and 2016 at two Boston academic hospitals, 99 patients (4.7%) had used cocaine [3]. The reported MI incidence in those with cocaine-related chest pain varies from 0.7% to 5.7% [4-6]. Animal studies have shown a direct, reversible depressant effect of cocaine on ventricular myocardium [7].

Acute cocaine usage can induce arterial constriction, thrombus formation, tachycardia, hypertension, and increased myocardial oxygen demand, leading to cardiovascular conditions, including ischemia, myocarditis, arrhythmias, stroke, and aortic dissection [8]. Chronic use accelerates atherogenesis and left ventricle (LV) hypertrophy, elevating the risk of ischemia, coronary artery aneurysm, and dilated cardiomyopathy [9]. There are competing arguments regarding the mechanism of cocaine cardiotoxicity. Cocaine use is associated with dilated cardiomyopathy, although a definitive cause-effect relationship remains uncertain. Patients with cocaine-induced dilated cardiomyopathy can present diversely, including heart failure symptoms, asymptomatic cardiomegaly, and manifestations linked to arrhythmias, conduction issues, thromboembolic events, or sudden death. The pathogenesis involves multiple factors, including direct cardiac toxicity, hyperadrenergic effects, potential infectious agents, and chronic ischemia. Cessation of cocaine intake may lead to rapid myocardial function recovery, except in cases of permanent injury from infarctions [10,11]. In a retrospective cohort that compared 738 cocaine users with heart failure with reduced ejection fraction (HFrEF) with matched non-cocaine users, cocaine consumption was linked to elevated risks of all-cause mortality, heart failure readmission, and overall readmission. Both nonselective and selective β-blockers could serve as safe treatment options for patients with heart failure and concomitant cocaine use [12].

Assessing heart failure in individuals with suspected cocaine consumption should adhere to the standard approach for evaluating heart failure in nonusers. This evaluation includes a comprehensive assessment, including medical history, physical examination, 12-lead ECG, chest radiographs, coronary imaging (such as CTA), trans-thoracic echocardiography, and serial monitoring of D-dimers and troponin T and I biomarkers. Recent cocaine ingestion can be confirmed through urine testing for cocaine and its metabolites. In this case, the patient experienced acute decompensated heart failure with a rapid recovery, which raises the question of the criteria and timing for diagnosing cocaine-induced heart failure. Currently, no specific timeframe exists delineating the onset of cocaine-induced heart failure. However, current guidelines follow the diagnostic approach recommended for reversible factors contributing to acute decompensated heart failure.

Follow-up

The patient did not come back for his one-month follow-up appointment. However, in a follow-up phone call, the patient reported to be in good condition with no active complaints.

## Conclusions

Cocaine-induced cardiomyopathy seems to have a rapid course of recovery. Accurate quantification of cases involving cocaine-induced cardiomyopathy and heart failure remains underestimated, with no well-defined clinical diagnostic criteria and management guidelines. There is a knowledge gap that necessitates further investigation and research.
